# Salvaging sepsis-associated atypical hemolytic uremic syndrome with terminal complement blockade: A case report

**DOI:** 10.1097/MD.0000000000047281

**Published:** 2026-01-23

**Authors:** Yuyang Qiu, Shen Zhou, Guanglin Huang, Yihong Gong, Guiyun Li, Wei Xie

**Affiliations:** a Department of Emergency, The Second People’s Hospital of Guiyang (Jinyang Hospital)/The Affiliated Jinyang Hospital of Guizhou Medical University, Guiyang, China.

**Keywords:** atypical hemolytic uremic syndrome, case report, eculizumab, sepsis, soluble C5b-9

## Abstract

**Rationale:**

Sepsis can trigger life-threatening atypical hemolytic uremic syndrome (aHUS) through dysregulated complement activation. Diagnosis is challenging due to overlapping features with septic shock, often leading to delayed recognition and high mortality.

**Patient concerns:**

A 23-year-old female with chronic kidney disease presented with a 3-day history of productive cough, fever, and progressive dyspnea, which rapidly escalated to respiratory failure, anuria, and coma within 24 hours.

**Diagnoses:**

Laboratory findings confirmed thrombotic microangiopathy (hemoglobin 57 g/L, platelets 69 × 10^9^/L, schistocytes 0.3%, lactate dehydrogenase [LDH] 469 U/L) and acute kidney injury (creatinine 535 µmol/L). The diagnosis of complement-mediated aHUS was established by markedly elevated soluble C5b-9 (sC5b-9) (578 ng/mL) with preserved a disintegrin and metalloproteinase with thrombospondin motifs 13 activity (89%).

**Interventions:**

Critical care included mechanical ventilation and continuous renal replacement therapy. Specific aHUS therapy comprised plasma exchange (10 sessions) and complement blockade with eculizumab (900 mg weekly for 4 weeks, then 1200 mg every 2 weeks), accompanied by meningococcal prophylaxis.

**Outcomes:**

By day 31, hematologic and renal parameters improved (platelets 72 × 10^9^/L, LDH 273 U/L, creatinine 151 µmol/L). By day 51, complement activity normalized (sC5b-9 261 ng/mL, a 54.8% reduction), with full hematologic recovery (platelets 198 × 10^9^/L, LDH 171 U/L), renal function near baseline (creatinine 96 µmol/L), and resolution of hemolysis.

**Lessons:**

In sepsis with persistent thrombocytopenia despite infection control, urgent sC5b-9 testing is critical to diagnose aHUS. Eculizumab, combined with supportive continuous renal replacement therapy and plasma exchange, can effectively reverse multi-organ failure even during active bacteremia, with serial sC5b-9 monitoring serving as a key biomarker for guiding therapy.

## 1. Background

Atypical hemolytic uremic syndrome (aHUS) represents a thrombotic microangiopathy (TMA) driven by dysregulation of the alternative complement pathway, culminating in microvascular thrombosis, hemolytic anemia, thrombocytopenia, and end-organ injury.^[1]^ Sepsis acts as a potent trigger in >40% of aHUS episodes, where bacteremia-derived pathogen-associated molecular patterns (e.g., endotoxin) directly activate *C3* convertase while inflammatory cytokines suppress complement regulatory proteins on endothelial surfaces.^[2]^ This convergence creates a vicious cycle of uncontrolled terminal complement activation, generating soluble C5b-9 (sC5b-9) that induces endothelial lysis and microthrombosis.^[3,4]^ Critically, this process often persists despite pathogen clearance, leading to multi-organ failure with acute mortality exceeding 25% and progression to end-stage renal disease in 50% of survivors.^[5]^

The diagnostic challenge lies in distinguishing sepsis-associated aHUS from septic coagulopathy. Overlapping features – thrombocytopenia, acute kidney injury, and lactic acidosis – delay diagnosis in >60% of cases, with critical therapeutic windows often missed beyond 72 hours.^[6]^ Biomarker-guided differentiation is essential: normal a disintegrin and metalloproteinase with thrombospondin motifs 13 (ADAMTS13) activity (>67%) excludes thrombotic thrombocytopenic purpura (TTP), whereas sC5b-9 elevation >240 ng/mL signifies complement-mediated injury rather than disseminated intravascular coagulation (DIC).^[7–9]^ This paradigm underscores sC5b-9’s role as a keystone diagnostic tool in infection-triggered TMA.^[8,9]^

Conventional therapies like plasma exchange (PEX) achieve limited success, with only 37.7% hematological remission in sepsis-associated aHUS and 46% progressing to dialysis dependence within 1 year.^[5,10]^ Eculizumab, a monoclonal antibody inhibiting *C5* cleavage, revolutionized management by preventing membrane attack complex (C5b-9) formation.^[11]^ This case demonstrates how prompt complement blockade can reverse catastrophic multi-organ failure unmasked by sepsis. The patient’s written informed consent has been obtained for the publication of this case report and the accompanying images. Ethical approval by the Institutional Review Board of the Affiliated Jinyang Hospital of Guizhou Medical University (Approval No. JYYY-2025-WZ-17).

## 2. Case presentation

A 23-year-old female with systemic lupus erythematosus-associated chronic renal failure (maintained hemodialysis for 2 years, baseline creatinine 151 μmol/L) presented to the emergency department with a 3-day history of productive cough, fever (38.9°C), and progressive dyspnea. Within 24 hours, the patient presented with acute respiratory failure (sitting breathing, pink frothy sputum), anuria (<100 mL/4 hours), and impaired consciousness (Glasgow Coma Scale 8/15). Blood gas analysis indicated severe hypoxemia (PaO_2_/FiO_2_ = 80 mm Hg), X-ray films showed bilateral lung infiltration, elevated procalcitonin (5.09 ng/mL), and blood routine showed white blood cell count 11.5 × 10^9^/L. The systolic blood pressure dropped rapidly to 77 mm Hg, and norepinephrine was needed to maintain the blood pressure. The initial assessment confirmed septic shock, with the sequential organ failure assessment score of 12; the acute physiology and chronic health evaluation II score was 29, accompanied by severe pneumonia and acute heart failure, and invasive ventilator support treatment was given after tracheal intubation.

Diagnostic investigations revealed: during the course of the disease, the severity of the condition progressively worsened. Subsequently, TMA was identified: hemolytic anemia: hemoglobin 57 g/L (baseline 76 g/L), lactate dehydrogenase (LDH) 469 U/L (ref: 120–250), schistocytes 0.3% on peripheral smear, total bilirubin 100.1 μmol/L, direct bilirubin 62.4 μmol/L, and indirect bilirubin 37.7 μmol/L, the direct antihuman globulin test negative; thrombocytopenia: platelets 69 × 10^9^/L (ref: 125–350); and acute kidney injury: creatinine 535 μmol/L (baseline 180 μmol/L). Complement dysregulation consistent with aHUS was confirmed through an extended panel: ADAMTS13 activity was 89%, effectively excluding TTP; marked elevation of the terminal complement complex sC5b-9 to 578 ng/mL (ref: <320) confirmed ongoing terminal complement activation; hypocomplementemia was evident with *C3* at 0.38 g/L (ref: 0.9–1.8) and C4 at 0.07 g/L (ref: 0.1–0.4); testing for *anticomplement factor H (CFH*) antibodies was negative; and a genetic panel sent for analysis included the *CFH*, *CFI*, *CD46*, *C3*, and *CFB* genes; a variant of uncertain significance was identified in the *CFH* gene. Functional complement assays (CH50/AH50) were not performed during the acute management phase. The calculated International Society on Thrombosis and Haemostasis (ISTH) overt DIC score was 4 (platelet count < 100 × 10^9^/L, elevated D-dimer (implied by the clinical context of sepsis and TMA), fibrinogen level was not obtained, no prolonged prothrombin time (PT) or activated partial thromboplastin time (APTT), indicating probable overt DIC. Additional hemolysis markers revealed significantly decreased haptoglobin (<0.08 g/L) and elevated reticulocyte count (2.27%). Only 1 sputum culture return showed positive for *Stenotrophomonas maltophilia*, while the other 2 sputum cultures and 2 alveolar lavage fluid cultures were all negative. Four blood culture tests were conducted, and all results were negative. The diagnosis of severe pneumonia as the primary source of sepsis was based on clinical presentation (fever, cough, and dyspnea), radiological evidence of bilateral infiltrates, and elevated procalcitonin, despite the absence of bacteremia (all blood cultures were negative). A single sputum culture grew *S maltophilia*, which was considered a colonizer or a secondary airway inhabitant rather than the primary pathogen in the context of broad-spectrum antibiotic exposure and improving respiratory status.

Therapeutic interventions commenced sequentially: critical support: intubation, mechanical ventilation, vasopressors (started with a dose of norepinephrine at 0.3 μg/kg/min), continuous renal replacement therapy (CRRT) for fluid/uremic control, and other treatments include: controlling blood pressure, correcting heart failure, and controlling acute pancreatitis; infection management: empirical broad-spectrum antibiotics were initiated with meropenem (2 g intravenous (IV) every 8 hours), later combined with tigecycline. The total duration of antibiotic therapy was 14 days. De-escalation to piperacillin-tazobactam was performed upon clinical stabilization and significant radiographic improvement, indicating adequate source control; and TMA-directed therapy: plasma and red blood cells were transfused, (PEX: 1.5 plasma volumes daily × 10 sessions) and (intravenous immunoglobulin: 0.4 g/kg/day × 5 days), methylprednisolone 1 g intravenous (IV) × 3 days, tapered to prednisone 1 mg/kg/day. Eculizumab: 900 mg intravenous (IV) weekly × 4 doses, then 1200 mg biweekly. Meningococcal prophylaxis was initiated 72 hours before the 1st eculizumab dose with intravenous amoxicillin-clavulanate (1.2 g, every 8 hours). This was transitioned after 2 weeks to oral ampicillin (0.5 g, 3 times daily) for the duration of eculizumab therapy, ensuring continuous coverage. No infectious complications occurred during the period of terminal complement blockade.

**Figure 1.**
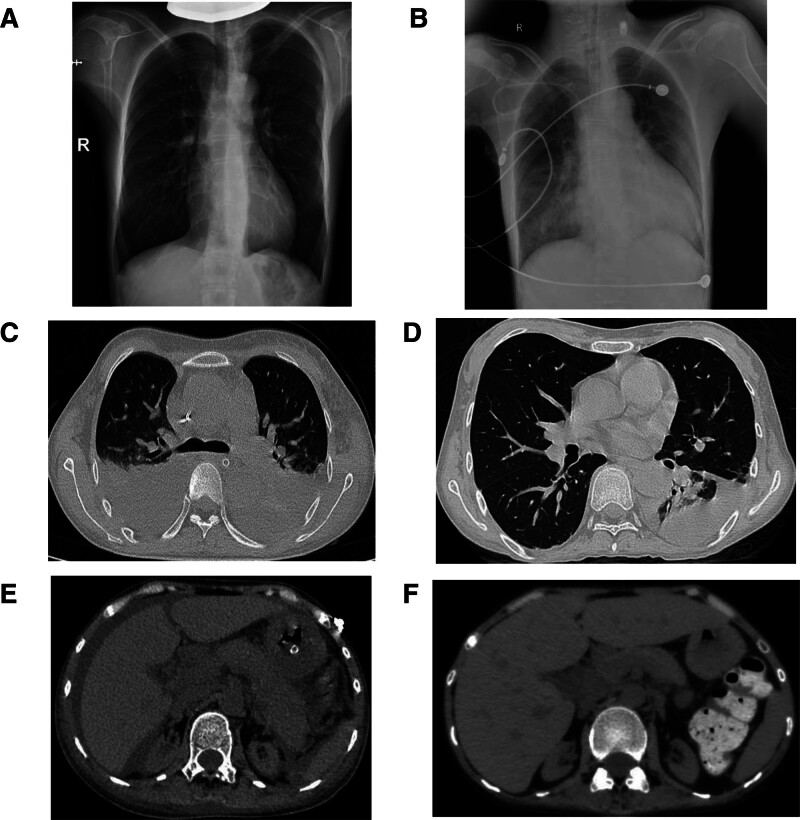
Comparison of radiological imaging results during the onset process of the patient. Panel (A) shows the X-ray taken on the 2 days before admission, nearly normal. Panel (B) shows the X-ray taken on the second day after admission, revealing multiple inflammations in both lungs (more obvious on the right side), accompanied by a small amount of pleural effusion on the left side. Panel (C) was taken on the 22nd day of admission. Severe inflammation occurred in both lungs, with increased pleural effusion on both sides, accompanied by incomplete dilation of the lower lobes of both lungs. Panel (D) was taken on the 42nd day of admission; the inflammation of both lungs had subsided compared with before, and both bilateral pleural effusion and hypoplasia of the lower lobes of both lungs improved compared with before. Panel (E) was taken on the 22nd day of admission. A large amount of fluid accumulation in the abdomen and pelvis can be observed, atrophy of both kidneys, exudation in the perirenal spaces of both kidneys, enlargement of the liver, and subcutaneous edema on both sides. Panel (F) was taken on the 42nd day of admission; effusion, inflammation, and edema were alleviated compared with before, but atrophy of both kidneys could still be observed.

Clinical course reflected treatment response: day 31, platelets increased to 72 × 10^9^/L, LDH decreased to 273 U/L, and creatinine decreased to 151 μmol/L. Day 51, complement activity normalized (sC5b-9 261 ng/mL, 54.8% reduction), ADAMTS13 activity was preserved (76%) without inhibitory antibodies, platelets increased to 198 × 10^9^/L, LDH decreased to 171 U/L, creatinine decreased to 96 μmol/L, the bilirubin level has dropped to the normal range and the hemolytic condition has been cured, without adverse or unanticipated events. The serial decline in sC5b-9, which corresponded with the weaning of vasopressors and liberation from mechanical ventilation, objectively demonstrated the reversal of terminal complement activation and paralleled the multi-organ recovery. Day 180, sustained renal recovery (creatinine 112 μmol/L), resumed renal dialysis treatment, without secondary infection. Chest computed tomography reevaluation showed resolution of bilateral infiltrates and pleural effusion (Fig. 1); serous cavity effusion is reduced compared with that at the onset of the disease, corroborating multi-organ recovery. At the 12-month follow-up, the patient remained dialysis-dependent, with a stable serum creatinine ranging between 110 to 120 μmol/L. No hematological relapse of aHUS has been observed, and she continues under regular nephrological review.

## 3. Discussion

Sepsis precipitates aHUS by creating a pro-inflammatory milieu that disrupts complement homeostasis. Bacteremia-derived endotoxins directly activate the alternative pathway by binding C3b and factor B, while cytokines like TNF-α suppress endothelial complement regulatory proteins.^[12]^ This dual assault culminates in uncontrolled *C5* convertase activity, generating sC5b-9 that lyses endothelial cells and initiates microvascular thrombosis.^[13]^ In our patient, sC5b-9 elevation to 578 ng/mL, which corroborated terminal complement activation as the driver of multi-organ failure.^[8,9,14]^ Notably, this cascade progressed despite antimicrobial therapy, underscoring that pathogen clearance alone cannot halt complement-mediated injury once amplified.^[12]^

Accurate TMA differentiation in sepsis hinges on strategic biomarker deployment. Normal ADAMTS13 activity (89%) excluded TTP, while undetectable Shiga toxin ruled out Shiga toxin-producing *Escherichia coli* (STEC)-HUS.^[8,9,14]^ Critically, sC5b-9 > 240 ng/mL distinguished complement-mediated TMA from sepsis-induced DIC, which typically exhibits normal complement profiles.^[7]^ This aligns with consensus guidelines advocating sC5b-9 as a pivotal test when TMA persists despite infection control.^[8,9,14]^ Genetic analysis further revealed a heterozygous *CFH* variant, illustrating how sepsis unmasks latent complementopathies.^[15]^

Eculizumab’s therapeutic value lies in its capacity to interrupt the terminal complement cascade irrespective of ongoing infection. With the treatment of eculizumab, we observed declining sC5b-9 (54.8% reduction) and platelet recovery to 198 × 10^9^/L, consistent with its rapid *C5* blockade mechanism.^[16]^ The synergistic use of CRRT and PEX proved critical: CRRT mitigated cytokine storm and uremia, while PEX temporarily reduced complement effectors before eculizumab achieved steady-state inhibition.^[5,11]^ The initial use of PEX in this case, before the confirmation of aHUS and initiation of eculizumab, reflected the diagnostic challenge in critically ill patients with overlapping features of TMA and septic shock. PEX was initiated as an empirical, potentially life-saving measure while awaiting definitive ADAMTS13 activity and complement studies, an approach sometimes considered in settings of high clinical suspicion for TTP or severe TMA of unknown etiology.^[17]^ However, it is recognized that PEX has limited efficacy in complement-mediated aHUS and may remove subsequently administered eculizumab, potentially reducing its effective serum concentration.^[18]^ Once aHUS was confirmed biochemically (with significantly elevated sC5b-9 and normal ADAMTS13 activity), and eculizumab was initiated, PEX was promptly discontinued to avoid interference with the monoclonal antibody. This sequence aligns with current guidance favoring targeted complement blockade over PEX once a diagnosis of aHUS is established. Crucially, meningococcal prophylaxis (amoxicillin and clavulanate potassium) averted secondary infections without compromising pathogen clearance.^[19]^

These findings challenge the dogma that active sepsis contraindicates complement inhibition. Rather, eculizumab benefit in reversing microvascular injury outweighs theoretical infection risks when paired with antimicrobial stewardship.^[20]^ For critically ill patients, sC5b-9 serves as both a diagnostic beacon and a treatment response biomarker.^[21]^

The diagnostic odyssey and successful intervention in this case underscore the critical need for a structured, algorithmic approach to TMA in the critically ill. The initial step hinges on cultivating a high index of suspicion upon recognizing the classic triad of microangiopathic hemolytic anemia, thrombocytopenia, and acute kidney injury. Once TMA is suspected, a swift, multidisciplinary diagnostic process must be initiated to distinguish between its various causes, as therapeutic strategies differ profoundly. The most time-critical exclusion is that of TTP, given its high mortality without prompt PEX. The cornerstone of this distinction is the measurement of ADAMTS13 activity, with a level below 10% being highly diagnostic for TTP.^[17]^ However, as this result is often unavailable on time, empiric PEX may be necessitated by strong clinical suspicion. Concurrently, other secondary causes of TMA must be rigorously investigated and excluded. These include STEC-HUS, through appropriate microbiological testing; autoimmune disorders such as systemic lupus erythematosus via serological profiling; severe malignant hypertension; and drug-induced TMA.^[22]^ STEC-HUS was ruled out by repeated negative stool tests for Shiga toxin. A comprehensive medication review revealed no exposure to drugs known to cause TMA. Malignant hypertension was excluded based on the patient’s normotensive presentation requiring vasopressor support. Heparin-induced thrombocytopenia was considered unlikely due to the classic TMA triad and a low 4T score, and was not confirmed by specific anti-PF4/heparin antibody testing. It is only after these potential causes have been reasonably ruled out that a diagnosis of aHUS can be considered. aHUS is a diagnosis of exclusion, fundamentally driven by dysregulation of the alternative complement pathway.^[16]^ In scenarios like our case, where a potent complement trigger such as sepsis is present, the threshold for suspecting complement-mediated aHUS should be low. The decision to initiate terminal complement blockade with an agent such as eculizumab, while ideally supported by genetic or functional complement studies, is often a time-sensitive clinical 1 in the face of life-threatening disease. This systematic, exclusion-based pathway – from suspicion to exclusion of mimics to targeted therapy – is paramount for salvaging organ function and improving outcomes in this complex disease landscape.^[23,24]^ Furthermore, for special groups at high risk of multiple organ failure, such as elderly patients, more refined and personalized treatment strategies should be adopted.^[25]^

### 3.1. Strengths and limitations

This study provides novel mechanistic insights by demonstrating serial sC5b-9 dynamics as a real-time biomarker guiding eculizumab therapy in active sepsis – a scenario previously considered high risk for complement blockade. The multimodal intervention protocol (CRRT, PE, and eculizumab) offers translational value for managing fulminant TMA, with objective evidence of multi-organ recovery (renal function normalization, radiological resolution). However, limitations include single-center experience and incomplete cost-benefit analysis of resource-intensive therapies. Generalizability may be constrained by the patient’s genetic predisposition (*CFH* variant), though sepsis-triggered complement dysregulation remains broadly applicable to critical care settings.

## 4. Patient perspective

The patient provided her perspective on the illness and treatment course. She expressed profound gratitude for the life-saving intervention, stating, “I am deeply thankful to the medical team who did not give up on me. Seeing the rapid reversal of the most critical symptoms after starting the targeted treatment gave me hope.” She described the prolonged hospitalization as a “physical and emotional marathon,” but emphasized that “transparent communication from the doctors about the risks, especially the potential for serious infection, helped alleviate my anxiety and allowed me to be part of the decision-making.” Her experience underscores that aggressive therapy can be acceptable to patients when paired with empathetic communication and clear goal-setting, culminating in her decisive view: “I chose fighting for survival over fear of complications.”

## 5. Conclusions

Sepsis-associated aHUS is a distinct entity where uncontrolled complement activation propagates multi-organ failure independent of pathogen eradication. Prompt differentiation from other TMAs via ADAMTS13 and sC5b-9 testing enables life-saving intervention. Eculizumab – initiated alongside CRRT/PEX and stringent antimicrobial prophylaxis – can salvage patients even during active bacteremia, redefining risk stratification in critical care. Serial sC5b-9 monitoring provides a roadmap for treatment efficacy.

## Author contributions

**Conceptualization:** Yuyang Qiu, Shen Zhou, Wei Xie.

**Funding acquisition:** Yuyang Qiu.

**Methodology:** Yuyang Qiu.

**Data curation:** Yuyang Qiu, Shen Zhou, Guanglin Huang, Yihong Gong, Guiyun Li.

**Supervision:** Wei Xie.

**Writing – original draft:** Yuyang Qiu, Shen Zhou.

**Writing – review & editing:** Yuyang Qiu, Shen Zhou, Guanglin Huang, Yihong Gong, Guiyun Li, Wei Xie.
